# Budget impact of sequential treatment with first-line afatinib versus first-line osimertinib in non-small-cell lung cancer patients with common EGFR mutations

**DOI:** 10.1007/s10198-020-01186-9

**Published:** 2020-04-23

**Authors:** Lotte Westerink, Jelmer L. J. Nicolai, Carl Samuelsen, Hans J. M. Smit, Pieter E. Postmus, Ingolf Griebsch, Maarten J. Postma

**Affiliations:** 1 Department of Health Sciences, University Medical Center Groningen, University of Groningen, Groningen, The Netherlands; 2 Asc Academics Inc., New York, USA; 3 Boehringer Ingelheim BV, Alkmaar, The Netherlands; 4 Outcomes Analytica AS, Oslo, Norway; 5 Rijnstate Hospital Arnhem, Arnhem, The Netherlands; 6 Department of Pulmonary Diseases, University Medical Centre, Leiden, The Netherlands; 7 Boehringer Ingelheim International GmbH, Ingelheim, Germany; 8 Unit of Pharmacotherapy, -Epidemiology and -Economics, Department of Pharmacy, University of Groningen, Groningen, The Netherlands; 9 Department of Economics, Econometrics and Finance, Faculty of Economics and Business, University of Groningen, Groningen, The Netherlands; 10 12 East 49th Street, New York, NY 10017 USA

**Keywords:** Budget impact, Afatinib, Osimertinib, Treatment sequencing, I15, I18, I15, I18

## Abstract

**Background:**

The therapeutic landscape for non-small-cell lung cancer (NSCLC) patients that have common epidermal growth factor receptor (EGFR) mutations has changed radically in the last decade. The availability of these treatment options has an economic impact, therefore a budget impact analysis was performed.

**Methods:**

A budget impact analysis was conducted from a Dutch healthcare perspective over a 5-year time horizon in EGFR-mutant NSCLC patients receiving first-line afatinib (Gilotrif^®^) versus first-line osimertinib (Tagrisso^®^), followed by subsequent treatments. A decision analysis model was constructed in Excel. Scenario analyses and one-way sensitivity analysis were used to test the models’ robustness.

**Results:**

Sequential treatment with afatinib versus first-line treatment with osimertinib showed mean total time on treatment (ToT) of 29.1 months versus 24.7 months, quality-adjusted life months (QALMs) of 20.2 versus 17.4 with mean cost of €108,166 per patient versus €143,251 per patient, respectively. The 5-year total budget impact was €110.4 million for the afatinib sequence versus €158.6 million for the osimertinib sequence, leading to total incremental cost savings of €48.15 million.

**Conclusions:**

First-line afatinib treatment in patients with EGFR-mutant NSCLC had a lower financial impact on the Dutch healthcare budget with a higher mean ToT and QALM compared to osimertinib sequential treatment.

**Electronic supplementary material:**

The online version of this article (10.1007/s10198-020-01186-9) contains supplementary material, which is available to authorized users.

## Background

Lung cancer is the worldwide leading cause of cancer mortality [1]. Non-small-cell lung cancer (NSCLC) is the most common type with 40% of the patients presenting with adenocarcinoma [2] Approximately 10–20% of Caucasian patients with non-resectable lung adenocarcinoma have somatic mutations of the epidermal growth factor receptor (EGFR) gene. The therapeutic landscape for patients with advanced EGFR mutation-positive NSCLC (exon 19 deletion and L858R) has changed radically with EGFR tyrosine kinase inhibitors (TKIs) becoming the new first-line standard treatment instead of chemotherapy. Data from multiple phase III randomized clinical trials (RCTs) have shown that the first-generation EGFR TKIs, erlotinib or gefitinib, and the second-generation EGFR TKI afatinib improved progression-free survival (PFS) and quality of life (QoL) compared to standard platinum-based chemotherapy [3–9]. More recent trials suggested that second-generation EGFR TKIs (afatinib and dacomitinib) may be more effective than first-generation agents [10, 11]. Recently, a third-generation TKI, osimertinib, has shown improved PFS compared to first-generation TKIs [12].

Eventually, patients treated with first-line EGFR TKIs develop resistance to treatment. The majority of patients (50–70%) progressing on first- and second- generation TKIs, develop secondary mutations such as the T790M point mutation. For patients with T790M-positive tumors, osimertinib is an approved second-line treatment based on improved PFS compared to standard of care observed in the AURA3 study [13]. ESMO guidelines recommended T790M mutation testing in all patients progressing on first-line TKI treatment [2]. For patients progressing on first-line therapy without T790M mutation, guidelines described complex and heterogeneous molecular mechanisms of resistance, for example, MET or HER2 amplification, BRAF or KRAS mutations, and small cell transformation. For these patients, the current standard treatment is platinum-based doublet chemotherapy (PDC). When patients progress on first-line osimertinib, the most frequent resistance mechanisms are MET amplification (15%) and EGFR C797S mutation (7%) [14]. There are currently no targeted treatment options available after osimertinib and patients failing first-line osimertinib treatment will receive chemotherapy or best supportive care [15, 16].

There is increasing evidence suggesting that patients treated with first-line afatinib are well suited for subsequent treatment with osimertinib. In a retrospective pooled analysis of data from 34 patients with common sensitizing EGFR mutations treated in the LUX-Lung 3, 6 and 7 studies, the median time on sequential treatment with osimertinib after first-line afatinib was 31.5 months [17]; the majority of patients were treated with osimertinib in the third, or later, line setting. Furthermore, a global observational real-world study showed that the median time on treatment for sequential first-line afatinib followed by second-line osimertinib was 27.6 months [18].

Due to the high number of life years lost by early death, lung cancer has one of the highest burdens of disease in the Netherlands [19, 20]. The economic impact of a first-line treatment with afatinib followed by re-biopsy guided osimertinib therapy versus treatment of first-line osimertinib followed by subsequent chemotherapy has not been assessed in the Netherlands. In this study, the medical cost of treatment and the budget impact of these different treatment options for patients with common EGFR mutations were evaluated.

## Materials and methods

### Model design

**Fig. 1:**
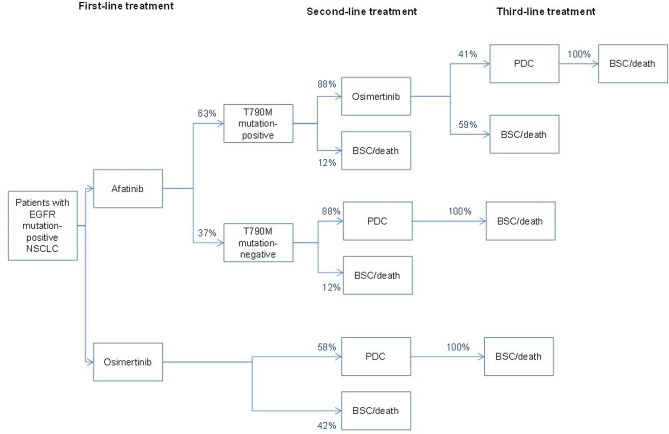
Decision-analytic model to assess the budget impact of first-line treatment with afatinib versus osimertinib in EGFR mutation-positive NSCLC patients. *BSC* best supportive care, *EGFR* epidermal growth factor receptor, *NSCLC* non-small-cell lung cancer, *PDC* platinum doublet chemotherapy

A decision-analytic model was constructed in Microsoft Excel to assess the budget impact of different first-line treatment approaches in advanced or metastatic NSCLC patients with common EGFR mutations. The model compared two treatment strategies (Fig. 1): first-line afatinib (Gilotrif^®^) followed by second-line osimertinib (Tagrisso^®^) (T790M positive on progression) or chemotherapy (T790M negative on progression) followed by third-line chemotherapy versus first-line treatment osimertinib followed by second-line chemotherapy. Patients are assumed to go into the health states best supportive care (BSC) or death after failing chemotherapy. The analysis was performed from the Dutch healthcare perspective. The model estimated outcomes and costs for each treatment approach over a 5-year period. This analysis was developed in accordance with the International Society for Pharmacoeconomics and Outcomes Research’s good practices for budget impact analysis [21].

This budget impact analysis represents a base case. One-way deterministic sensitivity analysis and scenario analysis are performed to determine the influence of uncertainty around model inputs.

### Model inputs

#### Study population

The eligible population included individuals with metastatic NSCLC whose tumors have EGFR deletion 19 or L858R mutations initiating first-line treatment. According to the Dutch Cancer Registration, there were 9175 patients with NSCLC in 2017 [20]. Approximately 10–20% of Caucasian patients with non-resectable lung adenocarcinoma have somatic mutations of the EGFR gene [2], leading to approximately 294 new patients every year in the Netherlands. It was assumed that a monthly mean of 24.5 new patients entered the model. A time horizon of 5 years was applied, in line with the decision-making context in the Netherlands. As this study is a budget impact analysis with a time horizon of 5 years, discounting was not applied.

#### Generation of dataset

To investigate the effect of treatment sequencing on time on treatment, a survival model with reconstructed individual patient time-to-event data was fitted using digitized PFS curves with data from controlled clinical trials. For afatinib as first-line treatment, patient level data from the LUX-Lung 3 clinical trial were used to fit the PFS curves [8]. In the LUX-Lung 3 trial, afatinib showed significant improvement with a median PFS of 11.1 months in comparison to 6.9 months of cisplatin plus pemetrexed in untreated advanced NSCLC patients with EGFR mutations [8]. To fit the PFS curves for the first-line treatment with osimertinib, data from the FLAURA clinical trial with untreated EGFR-mutated advanced NSCLC patients were applied. The FLAURA trial demonstrated a significantly longer median PFS of 18.9 months with osimertinib versus standard EGFR TKIs gefitinib and erlotinib with a median PFS of 10.2 months [12].

Eventually the majority of patients will develop resistance to first-line treatment, mostly by developing secondary mutations such as the T790M point mutation. Data from the AURA3 [13] and IMPRESS [22] studies was used for second-line osimertinib and chemotherapy, respectively. In the AURA3 trial, osimertinib showed a significant improvement in median PFS at 10.1 months versus 4.4 months for chemotherapy (pemetrexed plus either carboplatin or cisplatin) in T790 mutation-positive advanced NSCLC patients. Furthermore in second-line treatment with chemotherapy, the IMPRESS trial showed that continuation of a standard EGFR TKI did not prolong PFS in patients who received PDC as a subsequent line of treatment [22]. Both treatment arms showed a median PFS of 5.4 months. For this reason, there will be no continuation of EGFR TKI treatment when chemotherapy is the second-line treatment. Because there is limited data on the efficacy of third-line chemotherapy, we assumed similar efficacy to second-line chemotherapy.

The demographic characteristics of patients included in the RCTs of LUX-Lung 3, FLAURA, AURA3 and IMPRESS (published between 2015 and 2018) were well balanced [8, 12, 13, 22]. All patients included concerned adult patients with histological confirmed lung adenocarcinoma, advanced or metastatic stage IIIB and IV with mainly the following common EGFR mutations: exon 19 deletion and exon 21 L858R point mutation (further mutation details are provided in Tables 4–7 in the Supplementary Data). The median age in the RCTs ranged from 60 to 64 years old and the distribution of female ranged 54–65%. The distribution of race ranged 32–36% for white, 62–79% for Asian and 1–3% other. The studies LUX-Lung 3 and IMPRESS did not elaborate on the distribution between white and other, therefore a range of 21–28% concerns a combination of both for these studies. The WHO performance status 0 ranged from 40 to 41% and performance status 1 from 59 to 60%. A WHO performance status of 0 indicates that the patient is fully active and able to carry out all predisease activities without restrictions and a WHO performance status of 1 indicates that the patient is restricted in physically strenuous activity but is ambulatory and able to carry out work of a light or sedentary nature, such as light office work [12]. Only the WHO performance status was not taken into account in the AURA3 study. The distribution of patient’s history of smoking was 65–67% for never, 30–32% for former and 2–8% for current. In both the AURA3 and IMPRESS studies, it was unknown what the percentage of former and current smokers was.

To ensure that survival data for patients with similar characteristics were used in the model, the model and analyses were limited to patients with common mutations. A network meta-analysis (NMA) was performed, following a Bayesian framework with two designed and estimated networks. The description of the performed NMA is included in the Supplementary Data. WebPlotDigitizer was used to extract the Kaplan–Meier data from the publications, R version 3.5.1 and the SurvHE package was used to generate pseudo-individual patient-level data [23]. The partitioned survival model with a Weibull distribution for the common mutations estimated the proportion of a cohort in each state. The modeled PFS values were then used as proxy to estimate time on treatment for each intervention (Table 1).

**Table 1:** Overview of model parameters included in the budget impact model for sequential treatment with afatinib versus first-line osimertinib in NSCLC patients with common EGFR mutations

	Mean	Afatinib	Osimertinib		PDC	BSC/death	Source and assumptions
			First-line	Second-line			
**Transition probabilities—afatinib treatment arm**							
*T790M mutation-positive*							
First-line	–	63%	–	–	–	–	[25]
Second-line	–	–	88%	–	–	12%	Assumption
Third-line	–	–	–	–	41%	59%	[13]
Fourth-line	–	–	–	–	–	100%	Assumption
*T790M mutation-negative*							
First-line	–	37%	–	–	–	–	[25]
Second-line	–	–	–	–	88%	12%	[10, 13]
Third-line	–	–	–	–	–	100%	Assumption
**Transition probabilities—osimertinib treatment arm**							
First-line	–	–	100%	–	–	–	–
Second-line	–	–	–	–	58%	42%	[12]
Third-line	–	–	–	–	–	100%	Assumption
**Disutilities and incidences of grade ≥ 3 adverse events**							
Anaemia	− 0.07346	0.44%	1.08%	0.72%	6.31%	–	[27]
Diarrhoea	− 0.0468	14.41%	2.15%	1.08%	–	–	
Fatigue	− 0.07346	1.31%	0.72%	1.08%	12.61%	–	
Neutropenia	− 0.0897	0.44%	–	1.43%	18.02%	–	
Rash	− 0.03248	16.16%	1.08%	0.72%	–	–	
Dyspnea	− 0.07346	–	0.36%	1.08%	–	–	Assumption based on disutilities from Nafees et al. [27]
Leukopenia	− 0.0897	0.44%	–	–	8.11%	–	
Paronychia	− 0.03248	11.35%	0.36%	–	–	–	
Stomatitis	− 0.0468	8.73%	0.36%	–	0.90%	–	
**Health states**							
First-line	0.710	0.688	0.707	0.672	–	[8, 12, 13, 21, 26, 27]	–
Second-line	0.730	0.708	0.727	0.692	–	–	–
Third-line	0.620	0.598	0.617	0.582	–	–	–
**Simulated mean PFS from survival model**							
First-line	–	16.99	20.80	–	–	[8, 12]	–
Second-line	–	–	15.23	6.71	–	[13]	–
Third-line	–	–	–	6.71	–	[22]	–
**Costs**							
Treatment costs/month	–	€2440.15	€6181.33	€3679.04	–	[31, 35, 36]	–
T790M mutation testing	€157.02	–	–	–	–	[37]	–
**Adverse events costs**							
Anemia	€1945.59	€8.50	€20.92	€13.95	€122.69	–	[38]
Leukopenia	€1935.48	€8.45	–	–	€156.93	–	–
Neutropenia	€1399.53	€6.11	–	€20.06	€252.17	–	–
Diarrhea	€1500.97	€216.30	€32.28	€16.14	–	–	[39]
Rash	€87.99	€14.22	€0.95	€0.63	–	–	–
Dyspnea	€827.16	–	€2.96	€8.89	–	–	[40]
Stomatitis	€1330.85	€116.23	€4.77	–	€11.99	–	–
Fatigue	€83.63	€1.05	€0.58	€0.86	€10.14	–	[41]
Paronychia	€2.21	€0.25	€0.01	–	–	–	[36]
Total cost per treatment for adverse events	–	€371.11	€62.46	€60.54	€554.33	–	–

	Base case (range of sensitivity analysis)
**Input data for one-way sensitivity analysis**	
Prevalence T790M (%)	63 (52.5–73.0)
Subsequent treatment T790M mutation positive (% patients)	88.0 (70.4–100.0)
Subsequent treatment after first-line osimertinib (% patients)	58.0 (46.4–80.0)
Afatinib cost (€)	2440.15 (1952.12–2928.18)
Osimertinib cost (€)	6181.33 (4945.07–7417.6)
PDC cost (€)	3679.04 (2943.23–4414.85)
T790M test cost (€)	157.02 (125.61–188.42)
Afatinib AE cost (€)	371.15 (296.89–445.33)
Osimertinib first-line AE cost (€)	62.46 (49.97–74.96)
Osimertinib second-line AE cost (€)	60.58 (48.46–72.65)
PDC AE cost (€)	554.33 (443.46–665.2)

*AE* adverse event, *BSC* best supportive care, *PDC* platinum doublet chemotherapy, *PFS* progression-free survival

#### Transition probabilities

Total time in progression-free disease and post-progression treatment options per treatment plan were calculated using a decision tree combining survival data and prescription data (Fig. 1). Mean survival time from survival modeling was used to calculate the expected time on treatment for the different interventions. In addition, data from real-world studies were used in the model [24, 25]. Prevalence of detectable T790M rates depend on available diagnostic testing technology and have been reported to range from 50 to 70% [17, 18, 26]. For patients treated with afatinib, a 63% probability of a T790M mutation was used in the base case [25]. Estimates of the proportions of patients receiving subsequent therapy were taken from the literature. In the LUX-Lung trials, 71% of the intention-to-treat population and 88% of patients from countries with universal reimbursement received subsequent treatment (Table 1) [17]. In FLAURA, 58% of patients progressing on first-line osimertinib received subsequent therapy and in AURA III 41% of patients with T790M mutation-positive disease who progressed on second-line osimertinib received subsequent treatment [12, 13].

Due to limited availability of overall survival data for the modeled interventions, we have assumed that patients not receiving subsequent therapy following progression will continue to progress and receive best supportive care and/or die.

#### Utilities

To be able to include adverse events into the budget impact model, disutilities related to specific adverse events in patients with NSCLC [27] were combined with data from health-related quality of life (HRQoL) in patients with NSCLC in different health states [28] (Table 1). The model considered ten grade ≥ 3 adverse events associated with treatment. Assumptions were made for dyspnea, leukopenia, paronychia, stomatitis based on available data [27]. For example, the utility of leukopenia was assumed to be the same as the utility of neutropenia. Table 1 gives an overview of all (dis)utilities, sources and assumptions.

Comparing the utility in progression-free (PF) patients on first-line treatment with PF patients on second-line treatment resulted in a slightly higher but not clinically relevant mean utility in patients on second-line treatment [28]. One potential explanation for this could be that patients who move on to a higher line of treatment comprise a subset that is relatively fit [28].

#### Costs

An overview of all healthcare costs used in the model is shown in Table 1. The base-case analysis was the Dutch healthcare perspective; only direct medical costs were considered. The costs of both afatinib and osimertinib are based on the price for a monthly prescription. The mean cost for having grade 3 adverse events was calculated per patient for every specific treatment step, irrespective of time on treatment. In the model, every patient starting a specific treatment step will have a mean cost for grade 3 adverse events irrespective of time on treatment. The cost of a T790M mutation test was taken into account for all patients progressing on first-line afatinib, as this is necessary to be eligible for sequential treatment with osimertinib.

### Model outputs

The budget impact analysis estimated the costs associated with each treatment approach. Costs were reported as annual total costs for the population and as quality-adjusted life months (QALMs).

In accordance with the ISPOR guidelines for budget impact analysis [21], the robustness of model assumptions and uncertainty around specific parameters were tested in one-way sensitivity analysis. These variables included the prevalence of the T790M mutation, subsequent treatment ratio’s after positive T790M mutation test, treatment cost for afatinib, osimertinib or PDC, respectively, and adverse event costs for different treatments. Model parameters mostly varied by 20% with a maximum of 100% in the base-case value. Due to the high reported variance in detected T790M mutations and recent data pointing towards heterogeneity between the first- and second-generation TKIs in tumor clonality and development of resistance mechanisms, sensitivity analyses were also performed to investigate how variance in detected T790M rates affected the model outcomes [29]. Results were displayed on tornado diagrams, ranked from the most sensitive to least sensitive parameters.

Scenario analyses were performed to explore alternative assumptions in line with the suggestion of the ISPOR guidelines for budget impact analysis [21]. A scenario analysis was performed using the log-normal distribution for the fitted patient survival data (compared to the Weibull distribution used in the base case) (Scenario A). A second scenario analysis was performed using a higher, real-world estimate (73.1%) of the proportion of patients treated with afatinib that developed a T790M mutation at progression on first-line treatment (Scenario B) [24]. Also, in line with the ISPOR guidelines for budget impact analysis, we did not elaborate confidence intervals for the scenarios in a formal statistical manner. Notably, scenarios are designed with a specific purpose to inform the decision maker without the idea to potentially compare them statistically. The rules of inference have limited relevance to the decisions which budget impact models seek to inform and decisions in this context are based on the mean net benefits in the scenarios [30].

## Results

**Fig. 2:**
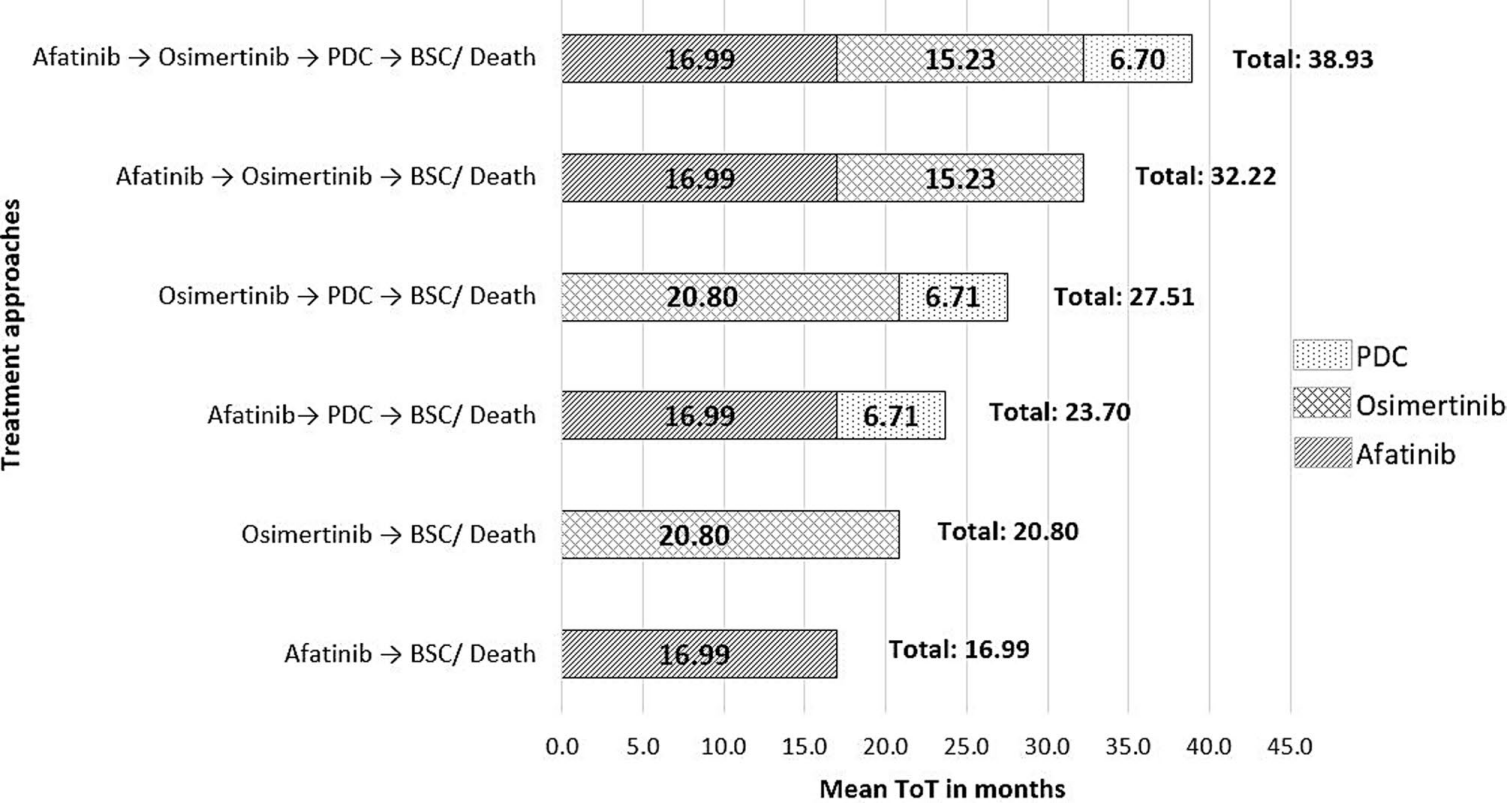
Outcomes survival analysis with Weibull distribution in decision-analytic model: mean total time on treatment for patients by treatment paradigms. *BSC* best supportive care, *PDC* platinum doublet chemotherapy

Outcomes survival analysis from the Weibull distribution in decision-analytic model for the various treatment paradigms is shown in Fig. 2. An overview of the resulting mean time on treatment from each treatment strategy, percentage of patients receiving subsequent therapies, as well as estimated QALM and total average costs per patient is shown in Table 1 in the Supplementary data. Overall, sequential treatment with first-line afatinib had a mean total time on treatment of 29.1 months, QALM of 20.2 with a mean cost of €108,166 per patient, in contrast to the treatment group with first-line osimertinib treatment that had a mean total time on treatment of 24.7 months, QALM of 17.4 for a mean cost per patient of €143,251. Treating with afatinib as first-line treatment would lead to an incremental time on treatment of 4.5 months and incremental QALM of 2.8, together with a lower cost of €35,085 for total mean cost per patient versus first-line treatment with osimertinib.

### Budget impact analysis

**Fig. 3:**
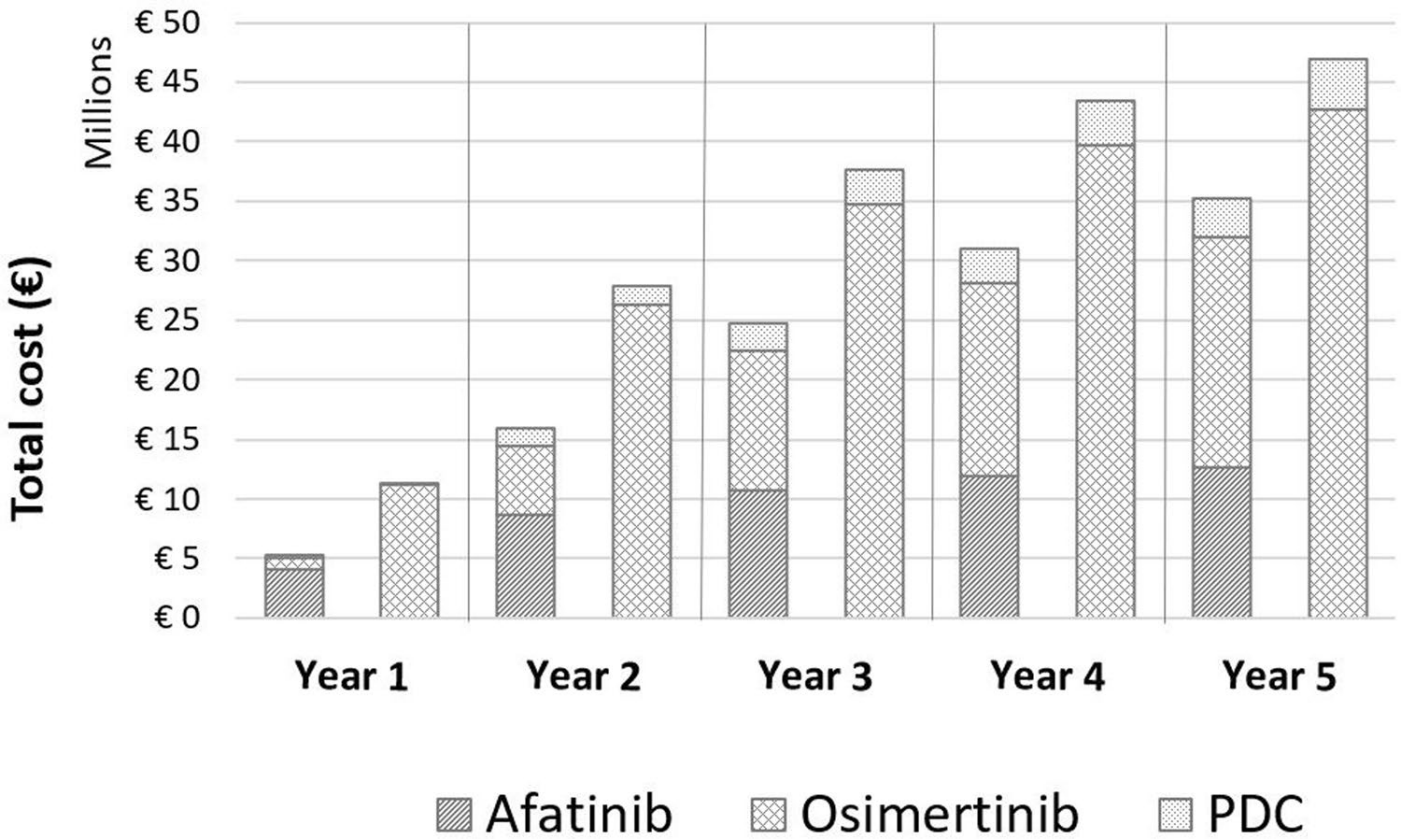
Total cost (€) per year for first-line treatment strategies afatinib versus osimertinib. *PDC* platinum doublet chemotherapy

Costs of each first-line treatment strategy were based on number of packages per year (a package is referred as one treatment cycle per patient; see appendix). The total budget impact of each treatment sequence is shown in Fig. 3. For the treatment sequence starting with afatinib, the total budget impact over 5 years was €110.4 million, with total costs per year ranging from €5.3 million in year 1 to €34.0 million for year 5. Introduction of osimertinib as second-line therapy in this strategy was responsible for 46% of the total cost in year 3 and increased proportionally over time.

For the treatment sequence starting with osimertinib, the total budget impact over 5 years was €158.6 million, with total costs per year ranging from €11.34 million in year 1, increasing to €36.98 million for the 3rd year, with lower incremental costs in years 4 and 5. The cost contribution of PDC to total costs was low reaching a maximum of 10% at year 5.

The incremental cost savings were €6.06, €12.25 million, €12.26 million, €9.87 million and €7.72 million for years 1, 2, 3, 4 and 5, respectively (see Appendix). Over a 5-year period of time, the incremental budget impact savings of sequential treatment with afatinib versus first-line treatment with osimertinib were estimated to be €48.15 million.

### Scenario analysis

Results of the two scenario analysis are presented in Table 2. Scenario A, with a log-normal distribution of the survival data showed a similar trend regarding budget impact outcomes to the base-case analysis using the Weibull distribution. The incremental budget impact over a 5-year period yielded cost savings of €54.97 million when using sequential treatment with afatinib in comparison to first-line osimertinib treatment. The second scenario analysis with 73.1% proportion of afatinib-treated patients receiving sequential treatment showed incremental budget impact cost savings of €41.34 million.

**Table 2:** Overview of outcomes for base case and scenario analyses of budget impact analysis in sequential first-line strategies afatinib versus osimertinib: number of claims, costs per year and budget impact

	Year 1	Year 2	Year 3	Year 4	Year 5
**Base case with Weibull distribution for the fitted patient survival data**					
**Number of claims**					
***First-line treatment strategy: start with afatinib***					
Afatinib	1656	3625	4491	4824	4941
Osimertinib	137	882	1851	2596	3030
PDC	69	338	576	728	815
***First-line treatment strategy: start with osimertinib***					
Osimertinib	1796	4276	5495	5940	6071
PDC	56	400	794	1,020	1,108
**Costs per year (€)**					
***First-line treatment strategy: start with afatinib***					
Afatinib	4,149,352	8,954,515	11,066,631	11,880,180	12,165,881
Osimertinib	852,936	5,471,444	11,470,829	16,078,865	18,767,065
PDC	270,545	1,289,728	2,183,376	2,750,702	3,064,386
Total costs	5,272,832	15,715,687	24,720,828	30,709,746	33,997,331
***First-line treatment strategy: start with osimertinib***					
Osimertinib	11,117,588	26,446,972	33,984,110	36,735,961	37,543,356
PDC	217,721	1,516,634	2,993,874	3,840,531	4,170,923
Total costs	11,335,309	27,963,606	36,977,985	40,576,492	41,714,309
**Budget impact (€)**					
Afatinib	4,149,352	8,954,515	11,066,631	11,880,179	12,165,881
Osimertinib	− 10,262,653	− 20,975,528	− 22,513,282	− 20,657,096	− 18,776,291
PDC	− 52,824	− 226,906	− 810,507	− 1,089,829	− 1,106,567
Total budget impact	− 6,062,477	− 12,247,919	− 12,257,157	− 9,866,746	− 7,716,987
**Scenario A: log-normal distribution for the fitted patient survival data**					
**Number of claims**					
***First-line treatment strategy: start with afatinib***					
Afatinib	1652	3498	4369	4839	5120
Osimertinib	140	944	1886	2617	3134
PDC	70	367	612	766	862
***First-line treatment strategy: start with osimertinib***					
Osimertinib	1802	4250	5622	6413	6899
PDC	54	410	774	997	1125
**Costs per year (€)**					
***First-line treatment strategy: start with afatinib***					
Afatinib	4,139,458	8,644,910	10,770,102	11,917,980	12,602,145
Osimertinib	876,051	5,857,182	11,687,707	16,211,293	19,405,814
PDC	276,751	1,395,414	2,314,444	2,888,088	3,233,655
Total costs	5,292,261	15,897,506	24,722,253	31,017,361	35,241,615
***First-line treatment strategy: start with osimertinib***					
Osimertinib	11,160,108	26,291,129	34,767,384	39,659,294	42,662,810
PDC	208,204	1,553,835	2,915,326	3,746,964	4,221,615
Total costs	11,368,311	27,844,964	37,682,710	43,406,257	46,884,425
**Budget impact (€)**					
Afatinib	4,139,458	8,644,910	10,770,102	11,917,980	12,602,145
Osimertinib	− 10,284,056	− 20,433,947	− 23,079,677	− 23,448,011	− 23,256,996
PDC	68,548	− 158,421	− 600,881	− 858,876	− 987,960
Total budget impact	− 607,651	− 11,947,458	− 12,910,457	− 12,388,897	− 11,642,810
**Scenario B: alternative proportion of afatinib treated patients receiving sequential treatment (73.1%)**					
**Number of claims**					
***First-line treatment strategy: start with afatinib***					
Afatinib	1656	3625	4491	4824	4941
Osimertinib	159	1023	2148	3012	3516
PDC	51	258	464	615	711
***First-line treatment strategy: start with osimertinib***					
Osimertinib	1796	4276	5495	5940	6071
PDC	56	400	794	1020	1108
**Costs per year (€)**					
***First-line treatment strategy: start with afatinib***					
Afatinib	4,149,352	8,954,515	11,066,631	11,880,180	12,165,881
Osimertinib	989,676	6,348,612	13,309,803	18,656,587	21,775,753
PDC	200,535	984,161	1,758,926	2,321,211	2,662,846
Total costs	5,339,563	16,287,288	26,135,360	32,857,978	36,604,479
***First-line treatment strategy: start with osimertinib***					
Osimertinib	11,117,588	26,446,972	33,984,110	36,735,961	37,543,356
PDC	217,721	1,516,634	1,993,874	3,840,531	4,170,953
Total costs	11,335,309	27,963,606	36,977,985	40,576,492	41,714,309
**Budget impact (€)**					
Afatinib	4,149,352	8,954,515	11,066,631	11,880,179	12,165,881
Osimertinib	− 10,127,912	− 20,098,360	− 20,674,307	− 18,079,373	− 15,767,603
PDC	− 17,186	− 532,473	− 1,234,948	− 1,519,320	− 1,508,107
Total incremental budget impact	− 5,995,746	− 11,676,318	− 10,842,624	− 7,718,514	− 5,109,829

*PDC* platinum doublet chemotherapy

### Sensitivity analyses

**Fig. 4:**
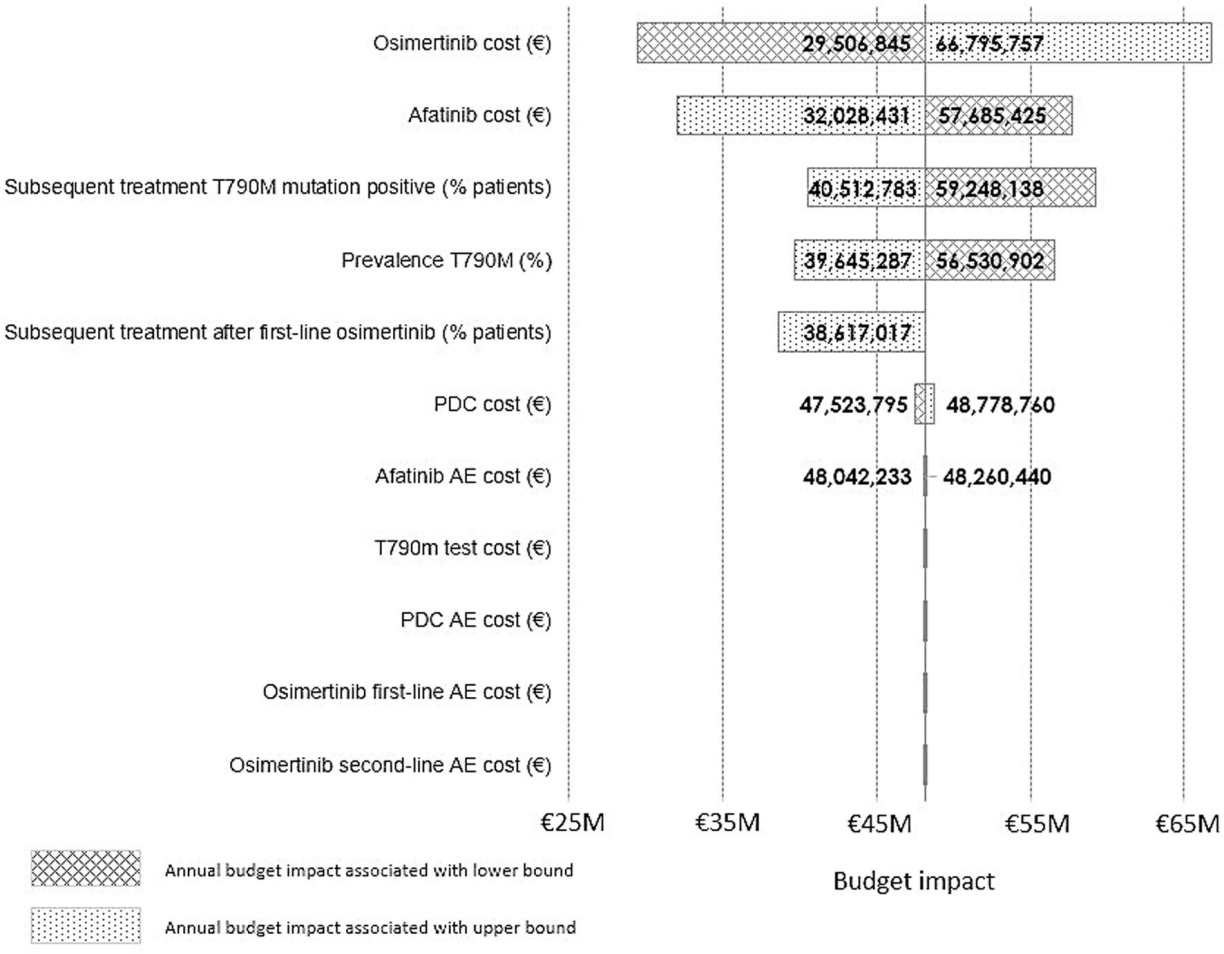
One-way sensitivity analysis: impact of changes of input parameters on the change in total annual budget in year 5. *AE* adverse event, *PDC* platinum doublet chemotherapy

Figure 4 shows the effect of key parameters on the total annual cost impact over 5 years. The results were most sensitive to the price of osimertinib and afatinib. Results were also sensitive to the percentage of patients with T790M mutation-positive disease receiving subsequent treatment, the percentage of patients testing positive for T790M mutations and the percentage of subsequent treatment after first-line therapy with osimertinib. The costs of adverse events for the different treatments and the cost of T790M mutation testing had only a minor influence on the incremental budget impact.

## Discussion

Over the last decade, various treatment options showed improved PFS data in clinical trials and will support improvement of the burden of disease for lung cancer. The availability of these treatment options has an economic impact, therefore a budget impact analysis was performed to estimate the impact of first-line sequential treatment of afatinib versus the introduction of osimertinib as first-line therapy for NSCLC patients with common EGFR mutations in the Netherlands. The results of this analysis indicated that the incremental budget impact savings of first-line sequential treatment with afatinib in comparison to first-line sequential treatment with osimertinib was €48 million over a period of 5 years. One-way sensitivity analysis showed the model was robust and the variables tested had no substantial influence on the incremental budget impact. As might be expected, the model was most sensitive to the price of osimertinib and afatinib. It is important to note that although there were differences in the tolerability of these agents, the increased frequency and costs of managing adverse events associated with afatinib were not associated with an increased overall treatment cost compared to osimertinib; afatinib had a lower total average cost per patient. Sensitivity analysis also showed that the costs associated with adverse events had little influence on the incremental budget impact.

The results of the model were also sensitive to the percentage of patients with T790M mutation-positive disease receiving subsequent treatment, the percentage of patients testing positive for T790M mutations and the percentage of subsequent treatment after first-line therapy with osimertinib. Indeed, the scenario analysis using a higher percentage for the proportion of patients that continued to receive second-line osimertinib following first-line afatinib resulted in smaller incremental budget impact savings due to a longer mean PFS time on second-line osimertinib and therefore increased drug costs. These results highlight the importance of T790M mutation testing, both for patient outcomes and assessment of treatment costs; further research is necessary to confirm the distribution and outcomes of T790M mutation testing. In addition, testing should already have been performed at the first sign of progression on first-line therapy to prevent deterioration of the performance status related to progressive tumor growth. More data on the subsequent treatment options after first-line osimertinib, as alternatives to PDC, are also needed as there is no evidence to support other treatment approaches at this time.

To our knowledge, this is the first full budget impact analysis of first-line sequential therapy with afatinib in comparison to first-line osimertinib and was based on the best currently available evidence, a validated budget impact model, and included sensitivity and scenario analyses. No prior analysis has compared the budget impact of afatinib to osimertinib in newly diagnosed patients with metastatic NSCLC whose tumors have EGFR deletion 19 or L858R mutations. Recently, the Dutch Healthcare Institute evaluated the budget impact of first-line osimertinib in NSCLC patients with EGFR mutations in the Netherlands over a 3-year horizon showing an estimated incremental budget impact of €18.6 million to €37.1 million [31]. This is broadly in agreement with our findings. It should be noted that this analysis considered different treatment paradigms to ours; the first-line osimertinib treatment strategy included third-line treatment with a PD-(L)1 inhibitor and the comparator arm included treatment with either a first- or second-generation TKI in the first-line setting and included a PD-(L)1 inhibitor third line after PDC or PDC third line after osimertinib second line. Our analysis did not include PD(L)1 inhibitors, as this treatment approach is not recommended by ESMO or Dutch guidelines for patients with EGFR-activating mutations [2, 32].

Further cost-effectiveness analysis is necessary to confirm our cost and quality-of-life assessment comparison between first-line sequential treatment with afatinib versus first-line treatment with osimertinib. The cost-effectiveness of osimertinib has previously been evaluated in Canada [33], the United States and Brazil [34]. In the analysis conducted in Canada, first-line osimertinib therapy was compared to EGFR TKIs gefitinib and afatinib and was not considered as a cost-effective treatment option at a willingness-to-pay threshold of $100,000 per QALY [33]. Similar results were reported in the United States and Brazil using World Health Organization cost-effectiveness threshold criteria; at current costs osimertinib was unlikely to be cost-effective in either the United States or Brazil [34]. The clinical benefit of first-line osimertinib over alternatives will likely carry the greatest weight in funding decisions [33].

There are several limitations that should be considered. As no data were available on the efficacy and median PFS of PDC as third-line treatment, data from the second-line setting of the IMPRESS trial were used which likely overestimated the treatment benefit in this treatment arm and also overestimated the additional costs coming from this treatment benefit. To avoid an over- or underestimation on the quality of life of treatments resulting from the exclusion of adverse events for which disutility data were not available, assumptions were made for dyspnea, leukopenia, paronychia and stomatitis. Further research regarding disutilities of adverse events is needed to support these assumptions. The intention of our model was to reconstruct the patients’ time on treatment per first-, second-, and third-line of treatment based on the clinical trials for afatinib, osimertinib and PDC, respectively. As no direct head-to-head studies have compared afatinib versus osimertinib, this model used a mixed treatment model approach. One of the largest differences between patient populations was the presence of the T790-positive mutation in all included EGFR-positive NSCLC patients in the AURA3 trial. The inclusion of these data had to be taken into account in our survival model to be able to reconstruct the effect of second-line treatment with osimertinib. The reason was that in the AURA3 trial the efficacy of second-line osimertinib was evaluated for this specific indication.

Even though the included RCTs were overall well balanced, we are aware of potential selection bias due to differences in patient populations such as age, race, smoking history, EGFR mutations status and presence of T790M positive mutation, resulting in potential over- or underestimation for both treatment arms.

The model did not include any other second- or third-line treatment options than PDC therapy or second-line osimertinib; it should be recognized that the inclusion of other emerging therapies would alter budget impact and total costs of both treatment strategies. Further research is needed with a head-to-head comparison of osimertinib versus afatinib and other second-generation TKIs in a clinical trial or real-world data setting to support the outcomes of this budget impact model. Finally, compliance was assumed to be 100% for all treatments which may not reflect real-world practice. This may overestimate treatment costs and would also affect the clinical benefits of treatment.

In conclusion, the use of afatinib as a first-line treatment approach would have a substantial lower financial impact on the healthcare budget in the Netherlands and would lead to a higher incremental time on treatment of 4.5 months and an incremental QALM of 2.8 compared to a sequential treatment approach starting with osimertinib. The total budget impact over 5 years was €110.4 million for the sequence starting with afatinib versus €158.6 million for the sequence starting with osimertinib, leading to a total incremental cost saving of €48.15 million. Together with clinical data, medicine prices and market access agreements will support governments and healthcare payers to decide how to manage the worldwide leading cause of cancer mortality.

## Electronic supplementary material

Below is the link to the electronic supplementary material.

Supplementary file1 (DOCX 220 kb)

## Data Availability

The data used in this research are not confidential since they were obtained from publicly available sources.
