# Supplementary material for: Budget impact of sequential treatment with first-line afatinib versus first-line osimertinib in non-small-cell lung cancer patients with common EGFR mutations
Source: Eur J Health Econ. 2020 Apr 23;21(6):931–43. doi: 10.1007/s10198-020-01186-9 (PMC7366569; doi:10.1007/s10198-020-01186-9)
Supplement: Supplementary file 1 — Supplementary file1 (DOCX 220 kb) [file 10198_2020_1186_MOESM1_ESM.docx]

**Budget Impact of sequential treatment with first-line afatinib versus first-line osimertinib in non-small-cell lung cancer patients with common EGFR mutations**

**Journal:** European Journal of Health Economics

**Authors:** Lotte Westerink, Jelmer LJ Nicolai, Carl Samuelsen, Hans JM Smit, Pieter E Postmus,

Ingolf Griebsch, Maarten J Postma

**Corresponding author:** Lotte Westerink^1,2^, [lotte.westerink@ascacademics.com](mailto:lotte.westerink@ascacademics.com)

1. Department of Health Sciences, University of Groningen, University Medical Center Groningen,

Groningen, The Netherlands

1. Asc Academics B.V., Groningen, The Netherlands.

**Supplementary data**

*Table 1: Overview of both treatment pathways with first-line afatinib sequential treatment versus first-line treatment with osimertinib*

|  | | **Proportion of patient population (%)** | **Mean ToT (months)** | **QALM** | **Total cost per patient (€)** |
| --- | --- | --- | --- | --- | --- |
| **Treatment approach: First-line treatment with afatinib** | | | | | |
| **T790M mutation positive** | **Afatinib 🡪 osimertinib 🡪 PDC 🡪 Death** | 22.7 | 38.9 | 26.7 | 161 456 |
|  | **Afatinib 🡪 osimertinib 🡪 Death** | 32.7 | 32.2 | 22.8 | 136 212 |
|  | **Afatinib 🡪 Death** | 7.6 | 17.0 | 11.7 | 41 834 |
| **T790M mutation negative** | **Afatinib 🡪 PDC 🡪 Death** | 32.6 | 23.7 | 16.3 | 67 235 |
|  | **Afatinib 🡪 Death** | 4.4 | 17.0 | 11.7 | 41 834 |
| **Treatment approach: First-line treatment with osimertinib** | | | | | |
| **Osimertinib 🡪 PDC 🡪 Death** | | 58.0 | 27.5 | 19.3 | 153 853 |
| **Osimertinib 🡪 Death** | | 42.0 | 20.8 | 14.7 | 128 609 |

*PDC: platinum doublet chemotherapy, ToT: Time on treatment, QALM: quality adjusted life month.*

**Network meta-analysis**

The network meta-analysis (NMA) used in this model was based on a previously published paper of Popat et al. [1] and is updated with data from LUX-Lung 7 and FLAURA [2, 3]. The same model was used to detect the relative performance in second-line using data from IMPRESS and AURA3 [4, 5].

The NMA was conducted using WinBUGS 1.4.1 (Medical Research Council Biostatistics Unit, Cambridge, UK) [6]. A Bayesian generalized linear fixed effects model was fit to calculate posterior distributions for the effects estimates, given likelihood functions derived from data and prior probabilities [7]. All baseline and intervention effect parameters were given flat (uninformative) normal density (0, 1000) prior and between-study standard deviation flat uniform distributions, with an appropriately large range given the scale of measurement. Only fixed-effects models were calculated in this simple analysis.

The model was run using 100 000 Monte Carlo simulations, 50 000 Monte Carlo runs were used as burn-in and discarded. The remaining 50 000 iterations were used for analysis. The model predicts a PFS hazard ratio (HR of 0.65; 95% credibility interval CrI: 0.46-0.90) in common mutations, (HR 0.62; CrI 0.41-0.62) for DEL19 and (HR 0.82; CrI: 0.48-1.29) in L858R for osimertinib versus afatinib in first-line. In second-line the model predicts a PFS hazard ratio is (HR 0.37; 95% CrI 0.29-0.48) in common mutations, (HR 0.34; CrI 0.25-0.47) in DEL19 and (HR 0.47; CrI 0.30-0.46) in L858R for osimertinib versus PDC.

Since the results of the network have been published elsewhere [1], only the results for afatinib, osimertinib and second-line PDC is reported here (Table 2, Table 3).

*Table 2: NMA outputs used to estimate relative performance in first-line treatment*

| **Mutation** | **Treatment** | **Mean** | **Standard**  **deviation** | **MC error** | **2.50%** | **Median** | **97.50%** |
| --- | --- | --- | --- | --- | --- | --- | --- |
| **Common** | **HR[Osimertinib vs. Afatinib]** | 0.65 | 0.11 | 0.00 | 0.46 | 0.64 | 0.90 |
| **Del19** | **HR[Osimertinib vs. Afatinib]** | 0.62 | 0.13 | 0.00 | 0.41 | 0.61 | 0.92 |
| **L858R** | **HR[Osimertinib vs. Afatinib]** | 0.82 | 0.21 | 0.00 | 0.48 | 0.79 | 1.29 |

*Table 3: NMA outputs used to estimate relative performance in second-line treatment*

| **Mutation** | **Treatment** | **Mean** | **Standard**  **deviation** | **MC error** | **0.025** | **Median** | **0.975** |
| --- | --- | --- | --- | --- | --- | --- | --- |
| **Common** | **HR[Osimertinib vs. PDC]** | 0.37 | 0.05 | 0.00 | 0.29 | 0.37 | 0.48 |
| **Del19** | **HR[Osimertinib vs. PDC]** | 0.34 | 0.06 | 0.00 | 0.25 | 0.34 | 0.47 |
| **L858R** | **HR[Osimertinib vs. PDC]** | 0.47 | 0.11 | 0.00 | 0.30 | 0.46 | 0.71 |

The data used for model inputs are presented here (Table 2-7) and in Figure 1. Since the included studies represent a mix of open label and double blinded trial designs, only independently reviewed PFS scores were included in the analyses. Please consult the original publication for sensitivity analyses on investigator versus independently reviewed PFS scores [1].

*Figure 1: Network diagram for the first-line treatment model*


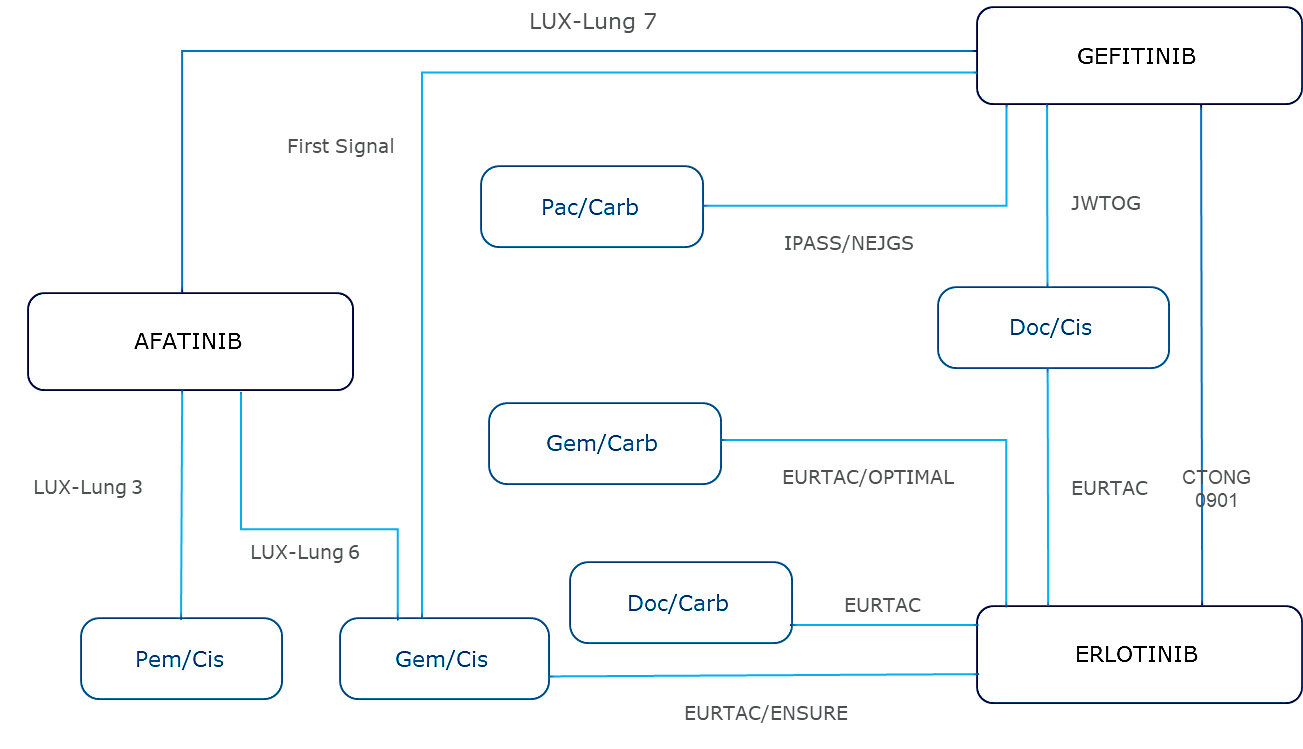


Table 4: Common mutations first-line EGFR

|  |  |  | **Mean** | **95% Confidence Interval** | |
| --- | --- | --- | --- | --- | --- |
| **Trial** | **Treatment A** | **Treatment B** | **Hazard Ratio** | **Lower** | **Upper** |
| Yang et al. 2012 (LUX Lung 3) | Afatinib | Pem/Cis | 0.48 | 0.35 | 0.66 |
| Wu et al 2013 (LUX-Lung 6) | Afatinib | Gem/Cis | 0.25 | 0.17 | 0.35 |
| Mok et al., 2009 (IPASS) | Gefitinib | Pac/Carb | 0.480 | 0.36 | 0.64 |
| Mitsudomi et al., 2010 (WJTOG3405) | Gefitinib | Doc/Cis | 0.490 | 0.34 | 0.71 |
| Maemondo et al., 2010 (NEJGSG002) | Gefitinib | Pac/Carb | 0.3 | 0.22 | 0.41 |
| Rossell et al., 2012 | Erlotinib | Doc/Cis | 0.47 | 0.28 | 0.78 |
|  | Erlotinib | Gem/Cis | 0.47 | 0.28 | 0.78 |
|  | Erlotinib | Doc/Carb | 0.47 | 0.28 | 0.78 |
|  | Erlotinib | Gem/Carb | 0.47 | 0.28 | 0.78 |
| Zhou et al, 2011 (OPTIMAL) | Erlotinib | Gem/Carb | 0.16 | 0.1 | 0.26 |
| Wu et al. 2015 (ENSURE) | Erlotinib | Gem/Cis | 0.42 | 0.27 | 0.66 |
| Han et al., 2012 (First SIGNAL) | Gefitinib | Gem/Cis | 0.54 | 0.27 | 1.1 |
| Park et al. 2015 (LUX-Lung 7) | Afatinib | Gefitinib | 0.74 | 0.57 | 0.95 |
| Soria et al. 2017 (FLAURA) | Osimertinib | Gef/Erlo | 0.45 | 0.36 | 0.57 |

Table 5: DEL19 First-line treatment for EGFR-mutations

|  |  |  | **Mean** | **95% Confidence Interval** | |
| --- | --- | --- | --- | --- | --- |
| **Trial** | **Treatment A** | **Treatment B** | **Hazard Ratio** | **Lower** | **Upper** |
| Yang et al. 2012 (LUX Lung 3) | Afatinib | Pem/Cis | 0.26 | 0.17 | 0.42 |
| Wu et al 2013 (LUX-Lung 6) | Afatinib | Gem/Cis | 0.2 | 0.13 | 0.33 |
| Mok et al., 2009 (IPASS) | Gefitinib | Pac/Carb | 0.380 | 0.26 | 0.56 |
| Mitsudomi et al., 2010 (WJTOG3405) | Gefitinib | Doc/Cis | 0.450 | 0.27 | 0.77 |
| Maemondo et al., 2010 (NEJGSG002) | Gefitinib | Pac/Carb | 0.35 | 0.23 | 0.52 |
| Rossell et al., 2012 (EURTAC, Tarceva prescribing information) | Erlotinib | Doc/Cis | 0.3 | 0.18 | 0.5 |
|  | Erlotinib | Gem/Cis | 0.3 | 0.18 | 0.5 |
|  | Erlotinib | Doc/Carb | 0.3 | 0.18 | 0.5 |
|  | Erlotinib | Gem/Carb | 0.3 | 0.18 | 0.5 |
| Zhou et al, 2011 (OPTIMAL) | Erlotinib | Gem/Carb | 0.13 | 0.07 | 0.25 |
| Wu et al. 2015 (ENSURE) | Erlotinib | Gem/Cis | 0.2 | 0.12 | 0.33 |
| Han et al., 2012 (First SIGNAL) | Gefitinib | Gem/Cis | 0.54 | 0.27 | 1.1 |
| Park et al. 2015 (LUX-Lung 7) | Afatinib | Gefitinib | 0.78 | 0.57 | 1.08 |
| Soria et al. 2017 (FLAURA) | Osimertinib | Gef/Erlo | 0.43 | 0.32 | 0.56 |

Table 6: L858R First-line EGFR

|  |  |  | **Mean** | **95% Confidence Interval** | |
| --- | --- | --- | --- | --- | --- |
| **Trial** | **Treatment A** | **Treatment B** | **Hazard Ratio** | **Lower** | **Upper** |
| Yang et al. 2012 (LUX Lung 3) | Afatinib | Pem/Cis | 0.75 | 0.48 | 1.19 |
| Wu et al 2013 (LUX-Lung 6) | Afatinib | Gem/Cis | 0.31 | 0.19 | 0.52 |
| Mok et al., 2009 (IPASS) | Gefitinib | Pac/Carb | 0.550 | 0.35 | 0.87 |
| Mitsudomi et al., 2010 (WJTOG3405) | Gefitinib | Doc/Cis | 0.510 | 0.29 | 0.9 |
| Maemondo et al., 2010 (NEJGSG002) | Gefitinib | Pac/Carb | 0.32 | 0.2 | 0.5 |
| Rossell et al., 2012 (EURTAC, Tarceva prescribing information) | Erlotinib | Doc/Cis | 0.55 | 0.29 | 1.02 |
|  | Erlotinib | Gem/Cis | 0.55 | 0.29 | 1.02 |
|  | Erlotinib | Doc/Carb | 0.55 | 0.29 | 1.02 |
|  | Erlotinib | Gem/Carb | 0.55 | 0.29 | 1.02 |
| Zhou et al, 2011 (OPTIMAL) | Erlotinib | Gem/Carb | 0.26 | 0.14 | 0.49 |
| Wu et al. 2015 (ENSURE) | Erlotinib | Gem/Cis | 0.54 | 0.32 | 0.9 |
| Han et al., 2012 (First SIGNAL) | Gefitinib | Gem/Cis | 0.54 | 0.27 | 1.1 |
| Park et al. 2015 (LUX-Lung 7) | Afatinib | Gefitinib | 0.66 | 0.44 | 0.99 |
| Soria et al. 2017 (FLAURA) | Osimertinib | Gef/Erlo | 0.51 | 0.36 | 0.71 |

Table 7: Common Mutation, DEL19, L858R Second-line EGFRM+

| **Trial** | **Treatment A** | **Treatment B** | **Hazard Ratio** | **Lower** | **Upper** |
| --- | --- | --- | --- | --- | --- |
| **Common mutations** | | | | | |
| Mok et al. 2017 (AURA III) | Osimertinib | Pem/Cis | 0.37 | 0.29 | 0.48 |
| Soria et al. 2016 (IMPRESS) | Gefitinib | Gem/Cis | 0.86 | 0.65 | 1.13 |
| **DEL 19** | | | | | |
| Mok et al. 2017 (AURA III) | Osimertinib | Pem/Cis | 0.34 | 0.24 | 0.46 |
| Soria et al. 2016 (IMPRESS) | Gefitinib | Gem/Cis | 0.76 | 0.54 | 1.11 |
| **L858R** | | | | | |
| Mok et al. 2017 (AURA III) | Osimertinib | Pem/Cis | 0.46 | 0.3 | 0.71 |
| Soria et al. 2016 (IMPRESS) | Gefitinib | Gem/Cis | 1.08 | 0.65 | 1.8 |

**References for Network Meta-analysis:**

1. Popat S, Mok T, Yang JC, Lungershausen J, Stammberger U, Griebsch I et al., Afatinib in the treatment of EGFR mutation-positive NSCLC–a network meta-analysis. *Lung Cancer*, 2014. 85(2): p. 230-238.

2. Soria JC, Ohe Y, Vansteenkiste J, Reungwetwattana T, Chewaskulyong B , Hyeong Lee K, Dechaphunkul A et al. Osimertinib in Untreated EGFR-Mutated Advanced Non-Small-Cell Lung Cancer. *N Engl J Med.* 2018; 378: 113-25.

3. Park K, Tan EH, O'Byrne K, Zahng L, Boyer M, Mok T et al. Afatinib versus gefitinib as first-line treatment of patients with EGFR mutation-positive non-small-cell lung cancer (LUX-Lung 7): a phase 2B, open-label, randomised controlled trial. *Lancet Oncol.* 2016; 17: 577-89.

4. Soria JC, Wu YL, Nakagawa K, Kim SW, Yang JJ, Ahn MJ et al. Gefitinib plus chemotherapy versus placebo plus chemotherapy in EGFR-mutation-positive non-small-cell lung cancer after progression on first-line gefitinib (IMPRESS): a phase 3 randomised trial. *Lancet Oncol.* 2015; 16: 990-8.

5. Mok TS, Wu YL, Ahn MJ, Garassino MC, Kim HR, Ramalingam SS et al. Osimertinib or Platinum-Pemetrexed in EGFR T790M-Positive Lung Cancer. *N Engl J Med.* 2017; 376: 629-40.

6. Lunn DJ, Tomas A, Best N, Spiegelhalter D. WinBUGS-a Bayesian modelling framework: concepts, structure, and extensibility. *Statistics and computing.* 2000; 10(4): 325-337.

7. Dias S, Ades AE, Welton NJ, Jansen JP, Sutton AJ. *Network meta-analysis for decision-making*. John Wiley & Sons Ltd., 2018
